# Precipitation and ectoparasitism reduce reproductive success in an arctic-nesting top-predator

**DOI:** 10.1038/s41598-018-26131-y

**Published:** 2018-06-04

**Authors:** Vincent Lamarre, Pierre Legagneux, Alastair Franke, Nicolas Casajus, Douglas C. Currie, Dominique Berteaux, Joël Bêty

**Affiliations:** 1 Département de biologie, chimie et géographie and Centre d’études nordiques, Université du Québec à Rimouski, 300 allée des Ursulines, Rimouski, QC G5L 3A1 Canada; 2 Arctic Raptor Project, Box 626, Rankin Inlet, NU X0C 0G0 Canada; 3 Department of Natural History, Royal Ontario Museum, 100 Queen’s Park, Toronto, ON M5S 2C6 Canada; 4 Present Address: CNRS-CEBC UMR 7372, Villiers en Bois, 79360 France

## Abstract

Indirect impacts of climate change, mediated by new species interactions (including pathogens or parasites) will likely be key drivers of biodiversity reorganization. In addition, direct effects of extreme weather events remain understudied. Simultaneous investigation of the significance of ectoparasites on host populations and extreme weather events is lacking, especially in the Arctic. Here we document the consequences of recent black fly outbreaks and extreme precipitation events on the reproductive output of an arctic top predator, the peregrine falcon (*Falco peregrinus tundrius*) nesting at the northern range limit of ornithophilic black flies in Nunavut, Canada. Overall, black fly outbreaks and heavy rain reduced annual nestling survival by up to 30% and 50% respectively. High mortality caused by ectoparasites followed record-breaking spring snow precipitation, which likely increased stream discharge and nutrient runoff, two key parameters involved in growth and survival of black fly larvae. Using the RCP4.5 intermediate climate scenario run under the Canadian Global Climate Model, we anticipate a northward expansion of black fly distribution in Arctic regions. Our case study demonstrates that, in the context of climate change, extreme weather events can have substantial direct and indirect effects on reproductive output of an arctic top-predator population.

## Introduction

Recent climate change has been associated with higher temperatures, and long-term projections indicate that an increase in the frequency of extreme weather events is likely^1^. These abiotic changes can have direct and/or indirect ecological consequences including trophic mismatch^2^, demographic effects^3^, and shifts in species distribution^4^ that generate new species assemblages^5^. Species’ range expansion can alter trophic relationships and contribute to novel host-parasite systems that can facilitate emergence of infectious diseases^6–8^ and increase host mortality.

Climate change has occurred at a faster rate in the Arctic compared to any other ecoregion globally^9^, resulting in increased temperature, melting permafrost and sea ice reduction^1,10^. Increased frequency of extreme weather events and wetter winters are also predicted for some arctic regions^11^. Warmer temperatures facilitate range expansion and establishment of pathogens and parasites in the Arctic^7,12,13^. For example, warmer conditions likely facilitated range expansion and establishment of a parasitic nematode of muskoxen *Ovibos moschatus* allowing the completion of the parasite life cycle in a single summer^12^. Emergence of diseases and parasites has been reported in arctic birds^13–15^ with potential interplay with changes in winter temperature affecting the host-parasite system in arctic-nesting birds^16^. Despite exposure and sensitivity of arctic host-parasite systems to climate change, empirical and model-based studies describing climate impacts on life history traits of parasites^17,18^ and fitness consequences on hosts^19^ in arctic regions are lacking^7^. This is surprising given that tundra ecosystems comprise low diversity of pathogens and parasites, relatively few species interactions^20^, and minimal anthropogenic disturbance, thus greatly facilitating understanding of the climate-driven mechanisms potentially affecting host-parasite interactions.

Here we examine the significance of ectoparasite outbreaks and heavy precipitation events on nestling survival in an arctic population of peregrine falcons monitored between 1982–1995 and 2008–2015 near Rankin Inlet, Nunavut, Canada (62°49′N, 92°05′W). Hematophagous ornithophilic black flies (Diptera: Simuliidae) are ectoparasites that can reduce breeding success in avian species by affecting adult nesting behaviour and increasing juvenile mortality^21^. Black fly outbreaks have rarely been implicated as a population-level source of mortality, however, the negative direct effects of increased frequency of heavy rain events on nestling survival is well established^3^ and partly explained the long-term decline in reproductive success in our study population^22^.

Our objectives were to: (i) illustrate the potential significance of ornithophilic black fly outbreaks at their northern range limit and heavy rain on nestling mortality in peregrine falcons; (ii) investigate the potential mechanisms (such as changes in temperature, precipitation and snow regimes) involved in promoting local black fly outbreaks and (iii) estimate the northern limit of black fly distribution using climate data (1990–2010) and project shifts in the northern limit using Canadian Global Climate Models with the RCP4.5 scenario^23^.

## Results

### Nestling survival and heavy rain

**Figure 1:**
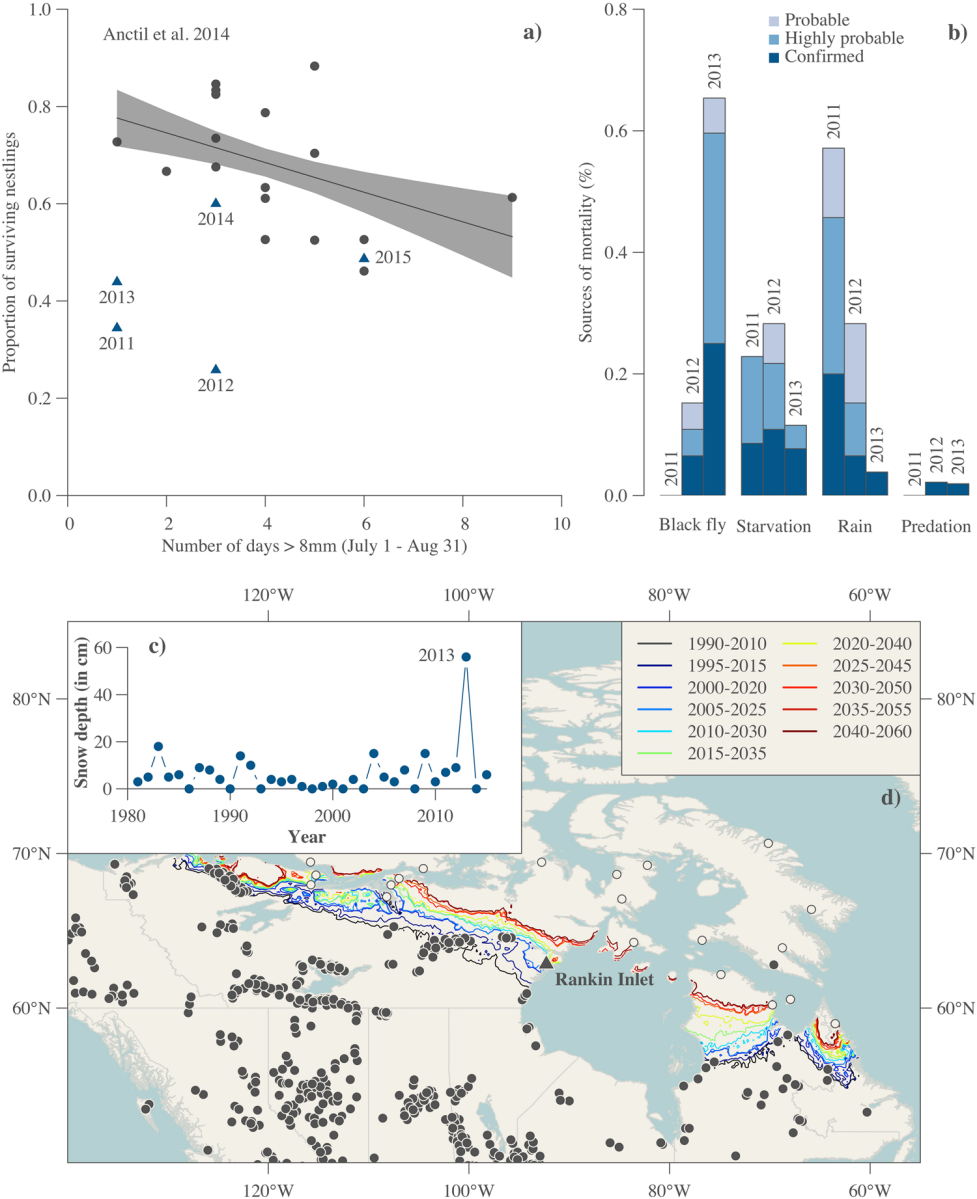
Identification of the climate-mediated mechanisms involved in peregrine falcon nestling mortality in the Canadian Low Arctic. (**a**) Relationship between the number of days with heavy rain (≥8 mm.day^−1^) recorded in July and August, and the proportion of peregrine falcon nestlings surviving up to 25 days old in the Rankin Inlet population (1982–1995 and 2008–2015). The solid black line and the shaded gray area correspond to the linear regression of Anctil *et al*.^3^ for the period 1982–1995 and 2008–2010 (black dots). We expanded the work of Anctil *et al*.^3^ by adding five extra years (2011–2015; blue triangles). (**b**) Contribution of black flies, starvation, rain and predation as causes of mortality in years with exceptionally low nestling survival (2011–2013). Additional (mostly unknown) causes of mortality are described in the Methods. The inner graph (**c**) indicates snow depth (cm) on the last day of May for the period 1981–2015 at Rankin Inlet. (**d**) Distribution map of ornithophilic black fly species in Canada. Dots refer to the presence or absence (filled and open dots respectively) of black flies. Black line represents the summer isotherm for the reference period (1990–2010) that best matches the northern limit of black flies (9.5 °C isotherm, see Methods). Color lines illustrate future potential predicted isotherms for 20-year periods from 1995 to 2060 based on the Canadian Global Climate Model (RCP4.5).

We first extended previously published data that describe the proportion of peregrine falcon nestlings surviving to 25 days by incorporating five additional years (2011–2015) from long-term population monitoring^3^. Nestling survival from 1982–1995 and 2008–2010 was negatively related to the number of days with heavy rain (≥8 mm.day^−1^) recorded in July and August (Fig. 1a). Four years were identified as outliers (1985: Studentized residuals = 2.40, P = 0.03; 2011, 2012 and 2013: all Studentized residuals ≤−2.46, all P = 0.02). A peak in microtine abundance likely explained the high productivity observed in 1985^24^. Despite relatively few days of heavy rain between 2011 and 2013, nestlings experienced lower survival (Fig. 1a). Difference between predicted survival based on long-term monitoring and observed survival indicate a reduction by up to 30–50% in nestling survival during these specific years (Fig. 1a).

### Causes of nestling mortality in extreme years (2011–2013)

Nestling mortality in 2011 was mostly due to a single heavy rain event with >19 mm precipitation in one day (Fig. 1a,b). Three episodes of heavy rain (10, 23 and 24 mm.day^−1^) caused high mortality in 2012 (Fig. 1a,b). Starvation mortality was observed in all years (Fig. 1b). Black flies represented a major source of nestling mortality over a very short period (<24 h) during the chick-rearing period only in 2013 (Fig. 1b). Evidence of nestling mortality from the biting effects of black flies was also found in 2012 (Fig. 1b), however the severity of this outbreak was much lower compared to 2013.

Variation in monitoring effort among nesting sites (i.e., presence of a camera; see Methods) prevented confirmation of the cause of nestling mortality at nesting sites that were not monitored using cameras (Fig. 1b). To address this, we compared mortality at wind-exposed and wind-sheltered nesting sites during the short period that encompassed the black fly outbreak. Black fly activity and abundance are negatively affected by wind velocity^25^ and we predicted that wind-exposed nesting sites would experience lower mortality compared to wind-sheltered nesting sites during the outbreak. We found that mortality was lower for nesting sites exposed to the prevailing wind (north oriented nesting sites) compared to sheltered ones during the outbreak in 2013 (χ^2^ = 3.84; P < 0.05, N = 27 nesting sites). The same analysis conducted prior to, and after, the outbreak showed no effect of exposure to the prevailing wind direction on mortality (χ^2^ < 1.3; P > 0.25; N = 30 and 23 nesting sites, respectively). Decreased mortality at wind-exposed nesting sites strongly suggests that blood-feeding black flies were probably responsible for deaths of nestlings at sites that were not monitored with cameras but that experienced a complete brood reduction during the 1-day black fly outbreak in 2013 (Fig. 1b).

### Weather, climate and black fly distribution

We examined weather variables that potentially explained high mortality rates associated with the 2013 outbreak. Analysis of weather variables available from 1981 to 2015 (temperature, precipitation and snow pack) indicated that snow depth recorded in late May 2013 was exceptionally high (56.0 cm in 2013 compared to 16.3 cm on average for the period 1981–2015; Fig. 1c)^26^ and was detected as an outlier (Studentized residuals = 9.89, P < 0.0001).

We constructed a distribution map of ornithophilic black fly species across North America from presence-absence data^27^, and estimated their northern distribution limit. The 9.5 °C summer isotherm of mean temperature for the months of June, July and August best matched the northern limit of the range of ornithophilic black fly species for the reference period (1990–2010). Forward projection of this summer reference isotherm by 20-year periods with five-year lags (e.g. 1995–2015, 2000–2020, 2005–2025, etc.) up to the period 2040–2060 predicted that ornithophilic black flies could become firmly established at latitudes consistent with our study area between 2010–2030 and 2015–2035 (Fig. 1d).

## Discussion

**Figure 2:**
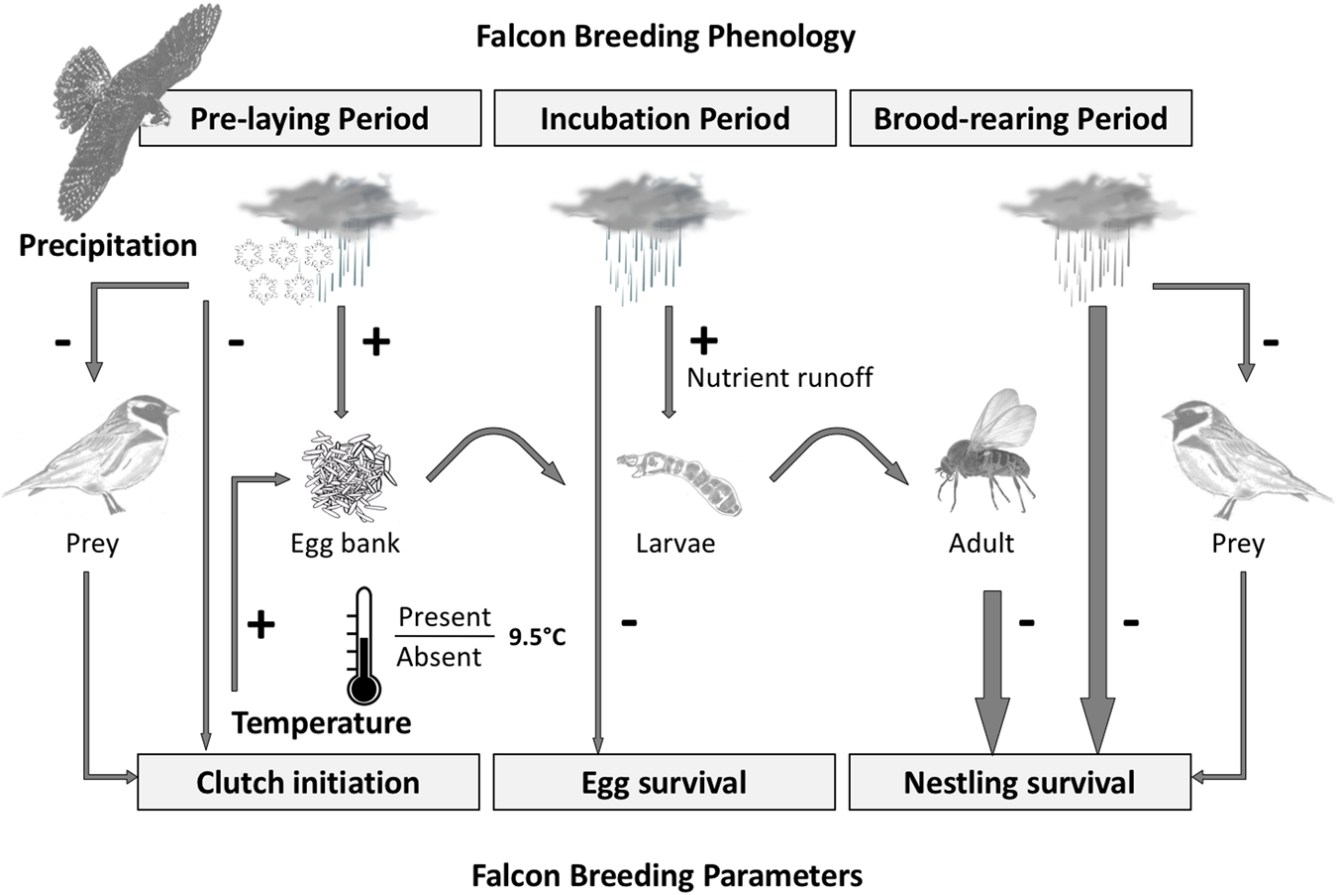
A qualitative assessment of direct and indirect effects of precipitation and temperature on reproduction of peregrine falcons nesting in the Canadian Low Arctic. Effects can be severe (wide arrows) or slight (narrow arrows) and occur during different phases of the breeding cycle. Curved arrows represent metamorphosis among life history stages of black flies. Mean summer temperature ≥9.5 °C likely reflects the isotherm at which populations of ornithophilic black fly species can become firmly established (Fig. 1). The magnitude and signs of the presented effects are justified in the discussion section.

Arctic species, including parasites and hosts have evolved under limiting environmental conditions and therefore, their life history traits are sensitive to even small changes in climatic conditions^28^. High exposure and sensitivity without any particular high adaptive capacity^29^ make arctic vertebrates particularly vulnerable to parasites^12,19^ and extreme weather events^3,30^. Our results confirm that increased frequency of heavy precipitation can reduce reproductive output of arctic-nesting peregrine falcons directly (through exposure to cold, wet weather), and indirectly through the outbreak of black flies described here and elsewhere^31^. We qualitatively summarized the frequency and severity of these effects on the reproduction of arctic-nesting peregrine falcon during major phases of the breeding cycle (Fig. 2). Heavy precipitation can have negative direct effects at any stage of the reproductive cycle and result in reduced reproductive output (Fig. 2). The frequency of heavy precipitation has increased over the past three decades and partly explained the long-term decline in falcon reproductive success^3^. The frequency of severe black fly effects is currently extremely rare but may increase in the Arctic as a result of climate warming (Figs 1 and 2).

The species of black fly (*Metacnephia saskatchewana*) likely responsible for mortality of nestlings in 2013 had not been reported in Rankin Inlet prior to 2016^31^. However, other ornithophilic species have been reported in Rankin Inlet (*Simulium silvestre*) and other nearby communities; (*Simulium anatinum* and *Simulium annulus* at Arviat and Baker Lake, Nunavut, Canada)^27^. Black fly outbreaks may be a rarely occurring natural phenomenon, or may represent an emerging source of mortality in our peregrine falcon population that could be related to climate change (i.e., changes in precipitation regimes and temperature). However, predicting shifts in geographic distribution of parasite species within the food web is challenging, and requires notably climate-driven projection^18^. We found that the measured and anticipated increase in summer temperature represents a projection of climatic conditions required for black fly species to expand their current northern range limit. In our opinion, the record-breaking spring snow depth recorded in 2013 may have contributed to increased stream discharge and nutrient runoff, two key parameters involved in growth and survival of black fly larvae^32,33^. The link between water flow and black fly larvae growth and survival combined with the expected increase in temperature and the frequency of extreme weather events with climate change could lead to an increase in the frequency of arctic black fly outbreaks in the near future. Although establishment of ornithophilic black flies in the Arctic under warmer temperatures is a prerequisite for an outbreak to occur, the amount of running water in spring is likely the proximate driver that influenced the severity of an outbreak.

Our results also demonstrate that single extreme weather events can reduce nestling survival in arctic-nesting birds. This adds to the growing body of literature showing that extreme weather conditions can reduce reproductive success during different stages of the breeding cycle^34–36^. Repeated heavy rain events across the brood rearing period can have direct consequences on peregrine falcon nestling survival^3^. Peregrine falcon nestlings younger than three weeks of age are sensitive to hypothermia during heavy rain events because they are unable to thermoregulate without being brooded or without the presence of sibling^37^. We also found that a single ectoparasite outbreak (2013) or extreme event of heavy rain (2011) can also have substantial negative effect on annual reproductive success by reducing offspring survival. In 2013, ectoparasitism (likely an indirect effect of precipitation; Fig. 2) is more likely driving the observed low nestling survival that year while in 2012, a combination of heavy rain and black fly outbreak are responsible for the low reproductive success. During the outbreaks, the biting effect of black flies resulted in widespread mortality due to the effects hemorrhagic coalescent lesions on the head and body of affected nestlings^31^.

Spring precipitation during the pre-laying period directly reduces clutch initiation. Precipitation can also indirectly reduce reproductive output, likely through lower body condition^38^ due to low prey availability. Precipitation during the pre-laying period likely triggers the hydrological conditions^33^ and nutrient runoff levels^32^ required for black fly reproduction. This ultimately results in indirect nestling mortality once adult black flies emerged (this study) and begin blood feeding (i.e., the proximate cause of mortality) on nestlings^31^. During incubation, precipitation directly reduces egg survival (Franke, unpublished data). During the brood rearing period, nestling survival is negatively affected by the direct effects of precipitation^3^ and by the indirect effects mediated through availability of prey^39^. Those direct and indirect linkages are summarized in Fig. 2.

One limitation of our study is that high mortality caused by ectoparasite was detected only during one year of our long-term population monitoring study and occurred over a very short period (<24 h), thus preventing an overall analysis of the mechanisms that may drive black fly outbreaks. Note that little is known about the biology of northern black flies and their interactions with wild birds; however, evidence suggests that the influence is likely to be as severe as that observed in the poultry and exotic bird industries^21^. Deaths from massive attacks are known to occur from toxemic shock or withdrawal of excessive blood^27^. For example, the deaths of more than 240 chickens and 20 turkeys in southwestern Manitoba in a single day were attributed to black fly attacks^40^. Although the duration of the outbreak was very short at Rankin Inlet in 2013, it was recorded in real time. Moreover, the severity of this single outbreak was remarkable and was supported by analysis of nesting site orientation, which is known to affect reproductive performance of cliff nesting birds^41^. In our study, we found that north-facing nesting sites (i.e. wind exposed nesting sites) experienced lower mortality only during black fly outbreak. This result is further supported by findings that show reproductive output is usually lower on north-facing nesting sites due mainly to lower solar radiation and exposure to cold northerly wind and storms^41,42^.

This study is one of the first to quantify the impact of an ectoparasite at its northern limit on the survival of a host population and illustrates how extreme weather events and increased temperature can have direct and indirect impacts on arctic wildlife. Despite increased efforts to model direct and indirect impacts of climate change on host-parasite systems^17,18^, long-term ecosystem-based monitoring will be required to detect climate-driven effects on host-parasites systems in the Arctic^7,18^.

## Methods

### Population monitoring

The population of peregrine falcons breeding near Rankin Inlet, Nunavut, Canada (62°49′N, 92°05′W) has been monitored continuously since 1980. However, we limited our analysis to 22 years (1982–1995 and 2008–2015) as the number of nestlings to hatch was not recorded in other years^3^. This population represents the highest known density of breeding peregrine falcons in the Arctic and the second highest density recorded worldwide^43^. Within the Rankin Inlet study area that encompasses approximately 455 km^2^, most rocky outcrops are oriented along a northwest-southwest axis and, consequently, most nesting cliffs used by peregrine falcons face southwest^24^. Nest monitoring procedures are extensively described in Franke *et al*.^22^. Since 2008, we deployed motion sensitive cameras at nests from egg-laying until fledging to document breeding phenology (lay and hatch dates), reproductive behavior (e.g. incubation duration, food delivery) and causes of mortality. We programmed cameras to capture one to three images when triggered by motion, after which they remained insensitive to movement for 5–15 seconds. In addition, camera recorded one image every 15 minutes and we replaced memory cards and batteries every 5–10 days during the nestling-rearing period. At each nest visit during this period, we systematically recorded growth of nestlings by assessment of body mass. Continuous monitoring by motion sensitive cameras during the reproductive season allowed the detection of two black fly outbreaks in the population. The first outbreak (25 July 2012) was less severe than the second outbreak (20 July 2013)^31^. We found no evidence of black fly outbreak in any other year in which cameras were deployed at nest sites (2008–2015) and we did not report nestling mortality associated with black flies for the 1982–1995 period^31^.

### Nestling survival and heavy rain

We followed Anctil *et al*.^3^ and defined “heavy rain” as any precipitation event ≥8 mm.day^−1^ (Fig. 1a). We compared the proportion of peregrine falcon nestlings surviving up to 25 days of age in the Rankin Inlet population as a function of the number of days with heavy rain recorded in July and August in recent years (2011–2015) to the regression model based on long-term monitoring (1982–1995 and 2008–2010)^3^. Weather data were available from the Environment Canada weather station located centrally within the study area^26^. We identified years with extremely high or low survival using Studentized residuals to test for the presence of outliers^44^. In addition, we examined the specific causes of nestling mortality observed in years with low nestling survival.

### Causes of nestling mortality between 2011–2013

Mortality events were recorded and classified into one of the following causes: (Fig. 1b).

#### Black fly

During the 2013 black fly outbreak, mortality events from black fly at camera monitored nests (N = 10) were confirmed through inspection of images. Descriptions and pictures of nestling mortality related to black fly are available in Franke *et al*.^31^. Remaining active nests (n = 20) were not monitored by camera but were visited weekly (every 5–7 days) during the chick-rearing period. At these nests, mortality from black flies was classified as highly probable when the full brood disappeared (dead nestlings are presumably carried away and cached by the adults) or probable when the brood partially disappeared during the period encompassing the date of the black fly outbreak. Decreased mortality recorded at wind-exposed nesting sites during the 1-day outbreak also indicate that blood-feeding black flies were very likely responsible for deaths of nestlings at nesting sites that were not monitored with cameras but that experienced a complete brood reduction during the outbreak (see results). The same methodology was used to assign mortalities during black fly outbreak in 2012. No evidence of nestling mortality related to black fly occurred in other years.

#### Starvation

Nestlings with growth curves below average growth curves for peregrine falcon nestlings in our population^24^ that did not die during or soon (≤2 days) after a rainstorm or black fly outbreak were confirmed to have died from starvation^3^. Mortality from starvation was classified as highly probable for nestlings that did not die during or soon after a rainstorm or black fly outbreak, that were fed less than three occasions per day prior to mortality and for which information on growth was not available. Mortality events from starvation that would have normally been classified as highly probable were classified probable when the event was not recorded due to improper camera placement or recording. Hence, the relative importance of starvation may have been slightly overestimated.

#### Rain

At camera monitored nests, mortality events from exposure to rain were confirmed for nestlings dying during rain event that were fed on three or more occasions per day^3^. At camera monitored nests where information on food delivery rate was not available, mortality from rain was classified as highly probable if nestlings died during a rain event. Mortality due to rain at nests that were visited weekly but were not monitored by camera was classified as probable if an episode of heavy rain occurred between two nest visits.

#### Predation

Nestling predation in the Rankin Inlet population is marginal and accounted for <1% of annual nestling mortality between 2009 and 2015. Predation events reported in Fig. 1b were all confirmed by camera.

#### Other

Nestlings that died of known causes that were not related to black fly, rain, starvation or predation were classified as Other (N = 5). Mortality events that could not be assigned to any known causes and that were not recorded on camera were classified as Unknown (2011: N = 7, 2012: N = 16, 2013: N = 9).

### Nesting site exposure and mortality

We performed a chi-square test to compare nesting site mortality between wind-exposed and wind-sheltered nesting site during the 1-day black fly outbreak on 20 July 2013. During the outbreak, wind speed was approximately 30 km.hour^−1^ blowing from the north^26^. Nesting sites oriented between 270° and 90° (0° = north) and nesting sites with nest located on the very top of a cliff were classified as wind-exposed while nesting sites oriented between 90° and 270° were classified as wind-sheltered. We first compared mortality at sheltered and wind-exposed nesting sites before (N = 30 nesting sites) during (N = 27) and after (N = 23) black fly outbreak in 2013.

### Weather, climate and black fly distribution

Data on snow depth on the last day of May for the period 1981–2015 (Fig. 1c) were available from the Government of Canada^26^ (Fig. 1c,d). We also used the Studentized residuals measure to test the presence of outlier(s) following a Student’s t distribution for a linear regression between snow depth on the last day of May and year^44^. To relate the distribution of ornithophilic black flies to a projected temperature increase across the North American Arctic (Fig. 1d), we mapped the presence and absence of 17 species with a boreal, subarctic and arctic distribution across North America for the period 1823–2003^27^. We then identified the summer isotherm of mean temperature for June, July and August that best matched the northern distribution of black flies (i.e. the isotherm with minimal distance from the observed northern distribution limit of black flies) for the reference period (1990–2010). Global climate models are complex mathematical representations of the Earth’s climate system that incorporate physical processes such as atmospheric flux, ocean circulation, land surface and sea ice dynamics, snow cover, and permafrost. We used the CGCM model because it is centered in Canada, and is more likely to represent future climate scenarios in that country relative to GCMs centered in other geographic regions. To estimate the northern shift of the isotherm that best matched the northern distribution of black flies, the CGCM model was coupled with the Representative Concentration Pathway (RCP) 4.5^23^, an IPCC scenario available through CMIP5 multi-model ensemble^45^ that represents an intermediate greenhouse gas concentration. These data were interpolated at a 10 km × 10 km horizontal resolution and we then projected the potential summer isotherms by 20-year periods with a lag window of five years (e.g. 1995–2015, 2000–2020, 2005–2015, etc.) up to the period 2040–2060 to predict the expansion of the northern limit of black flies. The map (Fig. 1d) was produced using the open-source statistical software R (version 3.4.0 available at https://www.R-project.org/) with the use of the following R packages: sp^46,47^, raster^48^, rgdal^49^ and rgeos^50^.
